# Hypertriglyceridemia is associated with an increased risk of peripheral arterial revascularization in high‐risk statin‐treated patients: A large administrative retrospective analysis

**DOI:** 10.1002/clc.23241

**Published:** 2019-08-01

**Authors:** Peter P. Toth, Sephy Philip, Michael Hull, Craig Granowitz

**Affiliations:** ^1^ CGH Medical Center Sterling Illinois; ^2^ Johns Hopkins University School of Medicine Baltimore Maryland; ^3^ Amarin Pharma Inc Bedminster New Jersey; ^4^ Optum Eden Prairie Minnesota

**Keywords:** cardiovascular disease, peripheral artery disease, risk, triglycerides

## Abstract

**Background:**

Peripheral artery disease (PAD) is common, and although it is associated with cardiovascular (CV) morbidity, mortality, reduced quality of life, and increased health care burden, PAD data are relatively scarce. Elevated triglycerides (TG) are associated with and are a risk factor for PAD.

**Hypothesis:**

Large administrative retrospective data may provide further insight into the relationship between hypertriglyceridemia and peripheral arterial revascularization in high‐risk statin‐treated patients.

**Methods:**

This retrospective administrative claims analysis of the Optum Research Database included statin‐treated patients aged ≥45 years with diabetes and/or atherosclerotic CV disease enrolled in 2010 and followed for ≥6 months. Patients with TG ≥150 mg/dL were propensity score‐matched to a comparator cohort with TG <150 mg/dL and high‐density lipoprotein cholesterol >40 mg/dL (n = 23 181 in each cohort). A sub‐analysis was conducted in patients with TG 200‐499 mg/dL and a matched comparator cohort (n = 10 990). Clustered *P*‐values were calculated using a Cox proportional hazard model with cohort as the independent variable (α, 0.05).

**Results:**

Multivariate analysis showed a 37% higher rate of peripheral arterial revascularization in the elevated‐TG cohort vs the comparator cohort (hazard ratio [HR] 1.370, 95% confidence interval [CI] 1.263‐1.486; *P* < .001). Results in the high‐TG sub‐cohort were similar, with a 49% higher rate of revascularization vs the comparator cohort (HR 1.489; 95% CI, 1.348‐1.644; *P* < .001).

**Conclusions:**

This large administrative retrospective analysis of high‐risk statin‐treated patients showed that elevated TG (≥150 mg/dL) and high TG (200‐499 mg/dL) were significant predictors of peripheral arterial revascularization; this warrants further study.

## 1 INTRODUCTION

Epidemiologic, genetic, and clinical evidence show that elevated triglycerides (TG) and TG‐rich lipoproteins are associated with increased risk of atherosclerotic cardiovascular disease (ASCVD) and play a causal role in ASCVD development and progression.^1^, ^2^, ^3^, ^4^, ^5^ Elevated TG are a risk factor for peripheral arterial disease (PAD).^6^, ^7^ PAD is common and is associated with cardiovascular (CV) morbidity, mortality, reduced quality of life, limb loss, and increased health care burden. The prevalence of PAD is >200 million persons worldwide, with >40 million persons affected in Europe.^8^ In the United States, the prevalence of PAD is ≥6.8 million individuals, with >13 000 deaths reported in 2015 and > 100 000 hospital discharges reported in 2014.^9^ Data on PAD are relatively scarce compared with data on coronary artery disease.^10^ Patients at high risk for CV disease, with controlled low‐density lipoprotein cholesterol (LDL‐C) but elevated TG and PAD, are increasingly encountered in clinical practice due, in part, to the increased prevalence of insulin resistance and diabetes mellitus. More information on the prevalence, health burden, health care costs, and resource utilization associated with this population is needed to optimize management and reduce associated morbidity and mortality.

The purpose of this retrospective analysis of a large medical claims database was to evaluate the impact of elevated TG on risk of peripheral arterial revascularization in high‐risk statin‐treated patients.

## 2 METHODS

Details of the study design have been published previously.^11^, ^12^ This retrospective analysis used the Optum Research Database, which includes >160 million individuals. Patients were eligible if they were ≥ 45 years old, had documented diabetes and/or ASCVD, had ≥1 prescription(s) for statin therapy filled between January 1, 2010 and December 31, 2010, and had ≥6 months of baseline data prior to the first statin claim; patients were ineligible if they had niacin remaining on the index date from a recent prescription fill. Patients were followed for a period of ≥6 months beginning at the index date to the earliest of the following: end of the study (March 31, 2016), date of disenrollment from their insurance plan, or death.

Three study cohorts were assessed. Patients in the elevated‐TG cohort were required to have TG ≥150 mg/dL at their most recent laboratory visit prior to the index date; the high‐TG sub‐cohort was required to have TG 200 to 499 mg/dL, and those in the comparator cohort were required to have TG <150 mg/dL and high‐density lipoprotein cholesterol (HDL‐C) >40 mg/dL.

The primary endpoint was the frequency of major CV events (a composite of CV‐related death, non‐fatal myocardial infarction, non‐fatal stroke, coronary revascularization, or unstable angina) in the follow‐up period. Secondary endpoints were direct health care costs (in US$) and resource utilization in the follow‐up period. Other prespecified analyses included the effects of TG on the probability of freedom from peripheral arterial revascularization as reported here. The primary and secondary endpoints were reported elsewhere.^11^, ^12^

Statistical analyses were carried out as follows: all study variables were analyzed descriptively and reported for the overall study sample, as well as stratified and statistically compared by cohort. Means and SDs were provided for all continuous variables; descriptive techniques that account for the length of observation time, such as per patient per month, were used for analyses of direct health care cost and resource utilization. Statistical comparison tests included Rao‐Scott test and χ² test for categorical measures and *t* test and analysis of variance for continuous measures. Multivariate pre‐match analyses used a Cox proportional hazard model to calculate hazard ratios (HRs) for time‐to‐event analyses. Kaplan‐Meier analyses were used to calculate time‐to‐event probabilities. Clustered *P*‐values were calculated using a Cox proportional hazard model with cohort as the independent variable. A *P‐*value <.05 was considered statistically significant.

A propensity score analysis was used to create a matched comparator study cohort similar to the analysis cohort, but without elevated or high TG, by controlling for confounding relationships. Propensity score matching was performed using a greedy match algorithm.^13^ The procedure used attempts to match each case to a single control based on the first 8 digits of the propensity score, which was estimated using logistic regression, then 7 digits, etc., until a match was found. The closest available match, known as the nearest neighbor, was used. Ties were resolved randomly. A maximum allowed propensity score difference (ie, a caliper) of 0.01 between the matched case‐control pairs was imposed a priori. Once a match was found, it was not reconsidered and the control was removed from the available pool for matches. The final sample of cases that were successfully matched to the controls was retained for analysis. The final list of variables included in the propensity score model was determined following review of the pre‐matching descriptive analyses of patient characteristics and other pre‐index measures and included age; gender; insurance type; region; baseline direct medical cost; LDL‐C level relative to the median, if available; baseline use of statins, fibrates, or omega‐3 fatty acids; and the following diagnoses: ASCVD, diabetes, stroke, hypertension, renal disease, and PAD. Patients in the elevated‐TG cohort were matched in a 1:1 ratio to the comparator cohort. Those who were not matched were not included in the descriptive analyses.

## 3 RESULTS

Baseline demographic and clinical characteristics of the patient cohorts have been described previously.^11^, ^12^ Approximately, 1.6 million statin‐treated patients with at least 1 prescription claim for a statin were identified from the database. After inclusion and exclusion criteria were applied and patients were propensity score matched, the elevated‐TG cohort and the high‐TG sub‐cohort consisted of 23 181 patients and 10 990 patients, respectively, with matching numbers of patients in the respective comparator cohorts. Other than the expected difference in lipid levels due to cohort inclusion criteria in the elevated‐TG and high‐TG sub‐cohorts, there were some baseline differences from their respective matched comparators that were statistically significant, but we do not consider these differences to be clinically important (Table 1).^11^, ^12^ Fibrate usage at baseline was similar between the elevated‐TG cohort and its comparator (7.4% vs 7.2%, respectively, *P* = .131) and between the high‐TG cohort and its comparator (9.1% vs 8.8%, respectively, *P* = .034; previously reported values of 13% and 8% for the two groups, respectively,^11^ represented fibrate usage in the first 6 months).

**Table 1:** Patient demographics, characteristics, and baseline comorbidities^11^, ^12^

	Elevated‐TG^a^ n = 23 181	Comparator^a^ n = 23 181	*P*‐value	High‐TG^b^ n = 10 990	Comparator^b^ n = 10 990	*P*‐value
Age, mean (SD), years	62.2 (9.6)	62.6 (9.9)	<.001	61.7 (9.6)	62.2 (9.9)	<.001
Female, n (%)	11 518 (49.7)	11 467 (49.5)	.244	5433 (49.4)	5424 (49.4)	.769
Insurance type, n (%)						
Commercial	15 823 (68.3)	15 855 (68.4)	.461	7589 (69.1)	7571 (68.9)	.556
Medicare	7358 (31.7)	7326 (31.6)	.461	3401 (30.9)	3419 (31.1)	.556
Duration of follow‐up, mean (SD), months	41.4 (23.7)	42.5 (23.9)	<.001	41.3 (23.8)	42.1 (23.9)	.018
Baseline^c^ lipid profile, mean (SD), mg/dL						
TG	220.31 (77.4)	97.9 (28.9)	<.001	263.8 (60.2)	98.2 (29.2)	<.001
LDL‐C	104.6 (41.1)	100.9 (35.0)	<.001	106.1 (43.2)	101.7 (34.7)	<.001
HDL‐C	42.3 (10.2)	55.1 (12.2)	<.001	40.4 (9.3)	55.0 (12.4)	<.001
Total cholesterol	190.2 (46.6)	175.4 (38.8)	<.001	198.2 (47.9)	176.3 (38.6)	<.001
Non‐HDL‐C^d^	147.9 (44.2)	120.4 (36.5)	<.001	157.9 (45.2)	121.2 (36.3)	<.001
Baseline comorbidities, n (%)						
Diabetes	19 392 (83.7)	19 478 (84.0)	.017	9326 (84.86)	9375 (85.30)	.048
ASCVD	6915 (29.8)	6800 (29.3)	.009	3185 (28.98)	3141 (28.58)	.156
MI	495 (2.1)	411 (1.8)	.003	235 (2.14)	189 (1.72)	.020
Stroke	750 (3.2)	674 (2.9)	.005	349 (3.18)	323 (2.94)	.177
Angina	1225 (5.3)	1179 (5.1)	.284	571 (5.20)	554 (5.04)	.562
Coronary revascularization	600 (2.6)	506 (2.2)	.002	299 (2.72)	213 (1.94)	<.001
Peripheral artery disease	3384 (14.6)	3317 (14.3)	.104	1561 (14.20)	1550 (14.10)	.704
Heart failure	1258 (5.4)	1088 (4.7)	<.001	626 (5.70)	519 (4.72)	<.001
Atrial fibrillation	1133 (4.9)	989 (4.3)	.001	527 (4.80)	472 (4.29)	.070
Hypertension	18 346 (79.1)	18 375 (79.3)	.462	8678 (78.96)	8723 (79.37)	.106
Renal disease	2832 (12.2)	2782 (12.0)	.196	1322 (12.03)	1314 (11.96)	.767

*Note*: Rao‐Scott test was used for binary measures. Robust standard errors were used for continuous measures.

Abbreviations: ASCVD, atherosclerotic cardiovascular disease; HDL‐C, high‐density lipoprotein cholesterol; LDL‐C, low‐density lipoprotein cholesterol; MI, myocardial infarction; non‐HDL‐C, non‐high‐density lipoprotein cholesterol; TG, triglycerides.

a Elevated TG ≥150 mg/dL and matched comparator with TG <150 mg/dL and HDL‐C > 40 mg/dL.

b High TG 200–499 mg/dL and matched comparator with TG <150 mg/dL and HDL‐C > 40 mg/dL.

c Baseline period excludes index date.

d Calculated by subtracting HDL‐C result from total cholesterol. This value was not calculated unless patients had both HDL‐C and total cholesterol laboratory result in period.

**Figure 1:**
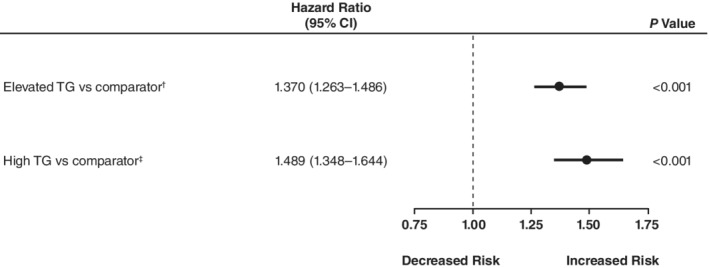
Effects of TGs on risk of peripheral arterial revascularization in statin‐treated patients with high cardiovascular risk*. *Multivariate analysis using Cox proportional hazard model. Separate pre‐match multivariate analyses of peripheral arterial revascularization were performed. Covariates included triglyceride cohort, as represented here, along with age (45‐54, 55‐64, ≥65 years), sex, insurance coverage type, geographic region of enrollment, baseline clinical characteristics (diabetes, atherosclerotic cardiovascular disease, low‐density lipoprotein cholesterol laboratory result in relation to median), and baseline medication use (fibrate, prescription omega‐3, both, and neither). ^†^Elevated triglycerides pre‐match cohort: TGs ≥150 mg/dL (n = 25 452 patients); comparator pre‐match cohort: TGs <150 mg/dL and high‐density lipoprotein cholesterol >40 mg/dL (n = 31 805 patients). ^ǂ^High TGs pre‐match cohort: TGs 200‐499 mg/dL (n = 12 364 patients); comparator pre‐match cohort: TGs <150 mg/dL and high‐density lipoprotein cholesterol >40 mg/dL (n = 31 805 patients). CI, confidence interval; TG, triglycerides

Analysis of the impact of hypertriglyceridemia on the risk of peripheral revascularization showed that freedom from peripheral arterial revascularization was significantly higher in the comparator cohort than in the elevated‐TG cohort (Table 2). At 5 years, the probability of peripheral arterial revascularization was 6.9% in the elevated‐TG cohort vs 4.9% in the comparator cohort. In a multivariate analysis controlled for patient characteristics and comorbidities, the rate of occurrence of peripheral arterial revascularization per unit time was 37% higher in the elevated‐TG cohort vs the comparator cohort (HR 1.370, 95% confidence interval [CI] 1.263‐1.486; *P* < .001; Figure 1). Similarly, in the high‐TG sub‐cohort, the comparator group had a higher rate of freedom from peripheral arterial revascularization than did the high‐TG sub‐cohort (*P* < .001) (Table 3). At 5 years, the probability of peripheral arterial revascularization was 7.3% in the high‐TG sub‐cohort vs 4.8% in the comparator cohort. Based on a multivariate analysis that controlled for patient characteristics and comorbidities, the rate of occurrence of peripheral arterial revascularization per unit time was 49% higher in the high‐TG cohort than in the comparator cohort (HR 1.489; 95% CI, 1.348‐1.644; *P* < .001; Figure 1).

**Table 2:** Freedom from peripheral arterial revascularization in statin‐treated patients with high cardiovascular risk and elevated triglycerides vs comparators (Kaplan‐Meier analysis)

Cohort	0.5 Year	1 Year	2 Years	3 Years	4 Years	5 Years	Clustered *P*‐value
Elevated triglycerides^a^	0.9873 (2 2795)	0.9783 (1 9265)	0.9637 (1 4678)	0.9525 (1 1723)	0.9420 (7942)	0.9308 (6096)	<.001
Comparator^b^	0.9913 (2 2884)	0.9849 (1 9566)	0.9752 (1 5149)	0.9652 (1 2319)	0.9574 (8514)	0.9512 (6664)	

Values represent probability of freedom from peripheral arterial revascularization (number of patients at risk). Clustered *P*‐value was calculated using Cox proportional hazard model with cohort as independent variable.

a Elevated triglycerides cohort: triglycerides ≥150 mg/dL.

b Comparator cohort: triglycerides <150 mg/dL and high‐density lipoprotein cholesterol >40 mg/dL.

**Table 3:** Freedom from peripheral arterial revascularization in statin‐treated patients with high cardiovascular risk and high triglycerides vs comparators (Kaplan‐Meier analysis)

Cohort	0.5 Year	1 Year	2 Years	3 Years	4 Years	5 Years	Clustered *P*‐value
High triglycerides^a^	0.9855 (1 0790)	0.9760 (9097)	0.9611 (6920)	0.9496 (5523)	0.9397 (3776)	0.9272 (2895)	<.001
Comparator^b^	0.9921 (1 0865)	0.9861 (9242)	0.9757 (7117)	0.9660 (5769)	0.9578 (3975)	0.9523 (3104)	

Values represent probability of freedom from peripheral arterial revascularization (number of patients at risk). Clustered *P*‐value was calculated using Cox proportional hazard model with cohort as independent variable.

a High triglycerides cohort: triglycerides 200‐499 mg/dL.

b Comparator cohort: triglycerides <150 mg/dL and high‐density lipoprotein cholesterol >40 mg/dL.

## 4 DISCUSSION

This large administrative retrospective study, which analyzed claims data from more than 45 000 statin‐treated patients in the Optum Research Database, showed that both elevated TG (≥150 mg/dL) and high TG (200‐499 mg/dL) were significant predictors of peripheral arterial revascularization (37% and 49% increased risk, respectively) in patients with high CV risk. These data support previous studies reporting an association between an atherogenic lipoprotein phenotype (elevated TG, reduced HDL‐C, and decreased LDL size) and increased risk of PAD.^14^, ^15^, ^16^

We previously showed that these elevated‐ and high‐TG patient populations had worse CV outcomes and higher health care resource use and direct costs than patients with TG <150 and HDL‐C > 40 mg/dL.^11^, ^12^ In patients with elevated TG and high TG, respectively, the overall risk of a major CV event was 26% and 35% higher than in the comparator cohorts after controlling for baseline characteristics and comorbidities (both *P* < .001).^11^, ^12^ Mean monthly direct health care costs were 11.8% higher in the elevated‐TG cohort than in the comparator cohort ($1438 vs $1270) and 15% higher in the high‐TG cohort than in the comparator cohort ($1462 vs $1279) (both *P* < .001).^11^, ^12^ The higher occurrence of an inpatient stay in patients with elevated TG (13% relative increase; 33.5% vs 30.5%) and high TG (17% relative increase; 34.0% vs 30.4%) (both *P* < .001) compared with matched controls would be expected to increase health care costs in these populations.^11^, ^12^

A strength of this study is that the data were obtained from a claims database encompassing a large number of patients drawn from actual clinical practice, and therefore the results generated from these data may be more reflective of actual use vs clinical trial evidence.^17^, ^18^ However, because the data were based on managed care health plan claims, they may not be generalizable to other health care delivery systems. Other limitations of claims data include potential data entry errors, missing data, and uncertainty about internal validity of data^19^; claims data also do not capture certain costs to patients, such as transportation and missed workdays. Importantly, because of the large sample size, small differences that have no clinical relevance may show statistical significance. This analysis was designed to assess the clinical and health economic burden of elevated TG despite generally controlled LDL‐C, and was not designed to assess the potential effects of any adjunctive therapy.

In conclusion, this large administrative retrospective study showed that both elevated (≥150 mg/dL) and high (200‐499 mg/dL) TG were significant predictors of the likelihood of peripheral revascularization in patients with high CV risk despite statin‐controlled LDL‐C. These results substantiate previous reports of the association of elevated TG with PAD^6^, ^7^ but also raise other questions. Statins have been shown to reduce the need for lower extremity revascularization.^20^, ^21^ The data presented here suggest that persistently elevated TG attenuate this statin benefit to a clinically significant degree. Further study of PAD patients is warranted to assess whether lowering TG will improve outcomes in statin‐treated patients with elevated TG and a history of diabetes or ASCVD, since they constitute a large population commonly encountered in clinical practice and because elevated TG are not routinely treated in patients who are either at risk for, or have, PAD.

## CONFLICT OF INTEREST

Peter P. Toth is a consultant and/or speaker for Amarin Pharma Inc., Amgen, Kowa, Novo‐Nordisk, Regeneron, and Sanofi. Sephy Philip and Craig Granowitz are employees and stock shareholders of Amarin Pharma Inc. Michael Hull is an employee of Optum. This study was sponsored by Amarin Pharma Inc, Bedminster, New Jersey, USA.
